# Prognostic potential of integrated morphologic and metabolic parameters of pre-therapeutic [^18^F]FDG-PET/CT regarding progression-free survival (PFS) and overall survival (OS) in NSCLC-patients

**DOI:** 10.1371/journal.pone.0307998

**Published:** 2024-07-29

**Authors:** Helena A. Peters, Daniel Weiss, Matthias Boschheidgen, Eduards Mamlins, Frederik L. Giesel, Georg Fluegen, Julian Kirchner, Gerald Antoch, Kai Jannusch

**Affiliations:** 1 Department of Diagnostic and Interventional Radiology, Medical Faculty and University Hospital Duesseldorf, Heinrich-Heine-University Düsseldorf, Düsseldorf, Germany; 2 Department of Nuclear Medicine, Medical Faculty and University Hospital Duesseldorf, Heinrich-Heine-University Düsseldorf, Düsseldorf, Germany; 3 Department of Surgery, Medical Faculty and University Hospital Duesseldorf, Heinrich-Heine-University Düsseldorf, Düsseldorf, Germany; 4 Center for Integrated Oncology Aachen Bonn Cologne Düsseldorf (CIO ABCD), Düsseldorf, Germany; Emory University, UNITED STATES OF AMERICA

## Abstract

**Purpose:**

This study aimed to evaluate the prognostic potential of pre-therapeutic [^18^F]FDG-PET/CT variables regarding prediction of progression-free survival (PFS) and overall survival (OS) in NSCLC-patients.

**Method:**

NSCLC-patients who underwent pre-therapeutic [^18^F]FDG-PET/CT were retrospectively analyzed. The following imaging features were collected from the primary tumor: tumor size, tumor density, central necrosis, spicules and SUV_max_. For standardization, an indexSUV_max_ was calculated (SUV_max_ primary tumor/SUV_max_ liver). Descriptive statistics and correlations of survival time analyses for PFS and OS were calculated using the Kaplan-Meier method and Cox regression including a hazard ratio (HR). A value of *p* < 0.05 was set as statistically significant. The 95%-confidence intervals (CI) were calculated. The median follow-up time was 63 (IQR 27–106) months.

**Results:**

This study included a total of 82 patients (25 women, 57 men; mean age: 66 ± 9 years). IndexSUV_max_ (PFS: HR = 1.0, CI: 1.0–1.1, *p* = 0.49; OS: HR = 1.0, CI: 0.9–1.2, *p* = 0.41), tumor size (PFS: HR = 1.0, CI: 0.9–1.0, *p* = 0.08; OS: HR = 1.0, CI: 0.9–1.0, *p* = 0.07), tumor density (PFS: HR = 0.9, CI: 0.6–1.4, *p* = 0.73; OS: HR = 0.3; CI: 0.1–1.1; *p* = 0.07), central necrosis (PFS: HR = 1.0, CI: 0.6–1.8, *p* = 0.98; OS: HR = 0.6, CI: 0.2–1.9, *p* = 0.40) and spicules (PFS: HR = 1.0, CI: 0.6–1.9, *p* = 0.91; OS: HR = 1.3, CI: 0.4–3.7, *p* = 0.65) did not significantly affect PFS and OS in the study population. An optimal threshold value for the indexSUV_max_ was determined by ROC analysis and Youden’s index. There was no significant difference in PFS with an indexSUV_max_-threshold of 3.8 (13 vs. 27 months; *p* = 0.45) and in OS with an indexSUV_max_-threshold of 4.0 (113 vs. 106 months; *p* = 0.40).

**Conclusions:**

SUV_max_ and morphologic parameters from pre-therapeutic [^18^F]FDG-PET/CT were not able to predict PFS and OS in NSCLC-patients.

## Introduction

Lung cancer (LC) is the second most commonly diagnosed cancer and is considered the main cause of cancer-related deaths accounting for 18% of all mortalities [1]. The 5-year survival rate is only 7% to 25% and the incidence is expected to further increase in most countries until the year 2035, making it a major public health challenge worldwide [2, 3]. Non-small cell lung cancer (NSCLC) accounts for the majority of LC diagnoses with approximately 85% [4]. For personalized clinical risk assessment and development of an ideal, patient-centered therapeutic strategy, accurate and comprehensive pre-therapeutic staging is of particular importance [5–7]. Imaging plays a pivotal role in the diagnostic work-up of patients with newly diagnosed LC. According to guidelines, ^18^F-fluoro-deoxy-glucose-positron emission tomography / computed tomography ([^18^F]FDG-PET/CT) has already proven to be an important tool for initial staging and detection of recurrence of small cell lung cancer (SCLC) and NSCLC [8]. This is mainly due to its high diagnostic accuracy in detecting loco-regional lymph node metastases- and distant metastases by using the combination of morphologic and functional imaging within the same imaging modality [9, 10]. Treatment decisions for LC patients are based on the patient’s general health condition and the TNM / Union for International Cancer Control (UICC) stage defined by [^18^F]FDG-PET/CT staging and/or additional histopathology [8]. Tumor stage is considered the most important prognostic parameter for clinical risk stratification, progression free survival (PFS), and overall survival (OS) [11, 12].

Although TNM- and UICC stages can be used as good prognostic predictors, there are patients within the same tumor stages, which have very different outcomes regarding PFS and OS. Thus, it is possible that patients after curatively intended R0 resection develop unexpected recurrence even in early tumor stages [13, 14]. In combination with the high mortality rate of LC patients, improving the pre-therapeutic risk stratification and early identification of patients with poorer prognosis may support a better and more patient-centered therapeutic decision making. The evaluation of the prognostic value of pre-therapeutic [^18^F]FDG-PET/CT has been the aim of several studies [6, 15, 16]. Agarwal et al. for e.g., showed that the preoperative SUV_max_ of LC primaries is not an independent predictor of survival in patients with early-stage NSCLC [17]. In contrast, other studies highlighted the SUV_max_ value as good prognostic marker for OS after LC resection [18–20]. In general, data on a potential prognostic benefit of SUV in NSCLC are heterogeneous which has also been highlighted by the review of Kitajima et al. [6]. Thus, further studies are needed to evaluate the prognostic potential of [^18^F]FDG-PET/CT also addressing morphological aspects of the primary tumor.

Therefore, this study aims to further elucidate the prognostic potential of pre-therapeutic [^18^F]FDG-PET/CT imaging markers with regard to PFS and OS at NSCLC-patients in order to improve patient-centered risk stratification and therapeutic decision-making.

## Materials and methods

### Patients

The study was approved by the institutional review board of the University Düsseldorf (study number: 2023–2331) and it was performed in accordance with the Declaration of Helsinki [21].

This retrospective study enrolled the data of 200 patients with the initial diagnosis of lung cancer at the University Hospital Düsseldorf. In accordance with the institutional review board, written informed consent was waived because of the retrospective study design. Only patients who met the following inclusion criteria were included for further data evaluation: (i) histologically confirmed NSCLC, (ii) pre-therapeutic [18F]FDG-PET/CT, (iii) UICC stages I to IV, (iv) no further malignancies, and (v) follow-up until at least 5 years after diagnosis.

### PET/CT imaging

[^18^F]FDG-PET/CT data were acquired on a Biograph mCT 128 (Siemens Healthineers, Erlangen, Germany). The average delay was 58 ± 2 min after injection of a bodyweight-adapted dosage of [^18^F]FDG (4 MBq/kg bodyweight). To ensure blood glucose levels below 150 mg/dL, blood samples were obtained, and patients needed to fast six hours prior to injection of [^18^F]-FDG. Mean activity was 253 ± 64 MBq. PET/CT was performed with a field-of-view from skull base to mid-thigh. All patients were placed in supine position with arms extended above the head. Weight-adapted iodinated contrast medium (Accupaque 300, GE Healthcare, Munich, Germany) was used in 61/82 patients. The CT component was started 70 seconds after intravenous injection of contrast agents. Automated tube current modulation was activated in all scans (presets 120 kV, 210 reference mAs, collimation 128 × 0.6 mm, pitch 0.8, slice thickness 5 mm). 21/82 patients received a low-dose [^18^F]FDG-PET/CT without contrast agents due to the availability of full-dose diagnostic CT prior to the PET/CT examination. A diagnostic low-dose lung scan in deep inspiration was additionally performed in full-dose and low-dose [^18^F]FDG-PET/CT for the CT-assessment of the lung tissue [22]. PET data were acquired for 3 min in each bed position (matrix size 256 × 256, axial field of view 21.8 cm and a Gaussian filter of 4 mm). Attenuation correction was performed and iterative reconstruction using ordered subsets expectation maximization was used with the following presets: 3 iterations and 21 subsets.

### Image analysis

TNM stage was determined according to the 8th version of the TNM classification for NSCLC for each patient by a board certified radiologists (senior physician) and nuclear medicine physician (senior physician) and confirmed by the institutional tumor board. Afterwards, TNM stage was transferred to the corresponding UICC stage.

**Fig 1:**
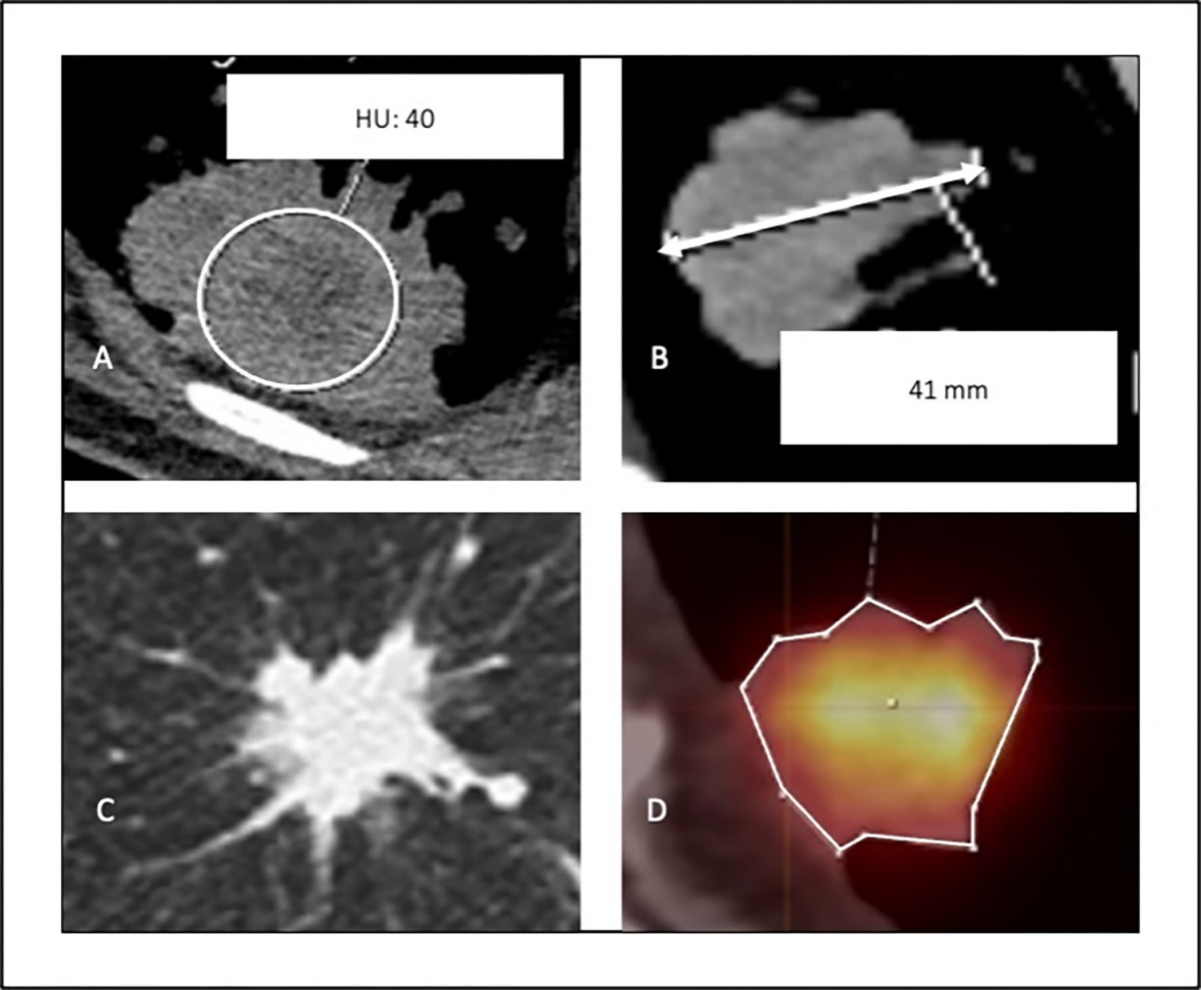
Example of image analysis. (**A**) calculation of density using a region of interest (ROI), (**B**) measuring of maximal diameters in mm, (**C**) evaluation of spicules and (**D**) measuring the SUV_max_ values in the tumor area with the highest metabolism using a volume of interest (VOI).

The following morphologic and metabolic data of the [^18^F]FDG-PET/CT examinations were collected for each patient: (i) maximal tumor size in mm, (ii) tumor density in Hounsfield Units (HU) using a size-adapted region of interest (ROI), (iii) visual appearance of spicules (yes/no), (iv) visual and measurable appearance of central necrosis (HU < 10), (v) SUV_max_ of the tumor area with the highest metabolic activity using an area adapted volume of interest (VOI). An example of data acquisition is visualized in Fig 1. Focusing on the tumor area with the highest metabolic activity, in analogy to existing studies, the collection of the SUV_mean_ was omitted [23, 24]. For standardization of SUV_max_ values the SUV_max_ value of liver parenchyma was measured for each patient in a pre-defined region and a ratio of the SUV_max_ of the primary towards the SUV_max_ of the liver parenchyma was calculated (indexSUV_max_).

### Patients’ demographics/-characteristics and histopathological parameters

In addition to imaging data, demographic data, tumor stage (UICC), histopathologic data, start-/ endpoint of therapy, survival, and / or progression data were obtained from each patient. Survival/ progression data were collected during guideline-adapted clinical follow-up with a period of at least five years. Tumor progression was defined in accordance to current Response Evaluation Criteria In Solid Tumors (RECIST) criteria [25]. Histopathological data of the primaries were acquired by endobronchial ultrasound-guided trans-bronchial needle aspiration (EBUS-TBNA), CT-guided biopsy, or by primary surgery. A detailed overview of the collected information is given in Table 1.

**Table 1:** Overview of patients’ demographics/-characteristics and histopathological parameters.

Patients’ demographics/-characteristics/- histopathological parameters	Value
**Number of patients**	n = 82
**Age (years)**	
Mean ± SD	66 ± 9
**Gender**	
Female	n = 25
Male	n = 57
**Histological subtype**	
Adenocarcinoma	n = 49
Squamous-cell carcinoma (SCC)	n = 22
Adenosquamous carcinoma (ASC)	n = 3
Large cell neuroendocrine carcinoma	n = 6
(LCNEC)	
Not further classified	n = 2
**Tumor stage (TNM)**	
T1	n = 29
T2	n = 28
T3	n = 16
T4	n = 9
N0	n = 31
N1	n = 13
N2	n = 24
N3	n = 14
M0	n = 52
M1a	n = 2
M1b	n = 19
M1c	n = 9
**Tumor stage (UICC)**	
I	n = 13
II	n = 12
III	n = 27
IV	n = 30
**Treatment**	
surgery	n = 23
surgery and chemo- or radiation therapy	n = 10
chemo- and radiation therapy	n = 26
chemotherapy	n = 6
radiation therapy	n = 10
neoadjuvant chemotherapy and surgery	n = 7
**Progress within 5 years**	
Yes	n = 54
No	n = 28
**Death within 5 years**	
Yes	n = 17
No	n = 65

### Statistical analysis

SPSS Statistics 26 (IBM Inc., Armonk, NY, USA) was used for statistical analysis. Descriptive analysis was performed, and data are presented as mean ± SD. For non-normally distributed continuous variables the median was reported including the interquartile range (IQR, 1st quarter - 3rd quarter). Mann-Whitney *U* test was used for an independent group comparison. In order to examine the correlation between [^18^F]FDG-PET/CT imaging parameters towards progression-free survival (PFS) and overall survival (OS), the Kaplan-Meier method was used. An optimal cut-off value for PFS and OS was determined by ROC-analysis and Youden’s index. Significance was calculated using the log-rank test. For PFS and OS, the median was expressed in months. Furthermore, cox regression and a hazard ratio (HR) were used to calculate the extent to which different [^18^F]FDG-PET/CT imaging parameters had a significant impact on PFS and OS. *P* values < 0.05 were statistically significant. For the hazard ratio, the corresponding 95% confidence interval (CI) was calculated.

## Results

**Fig 2:**
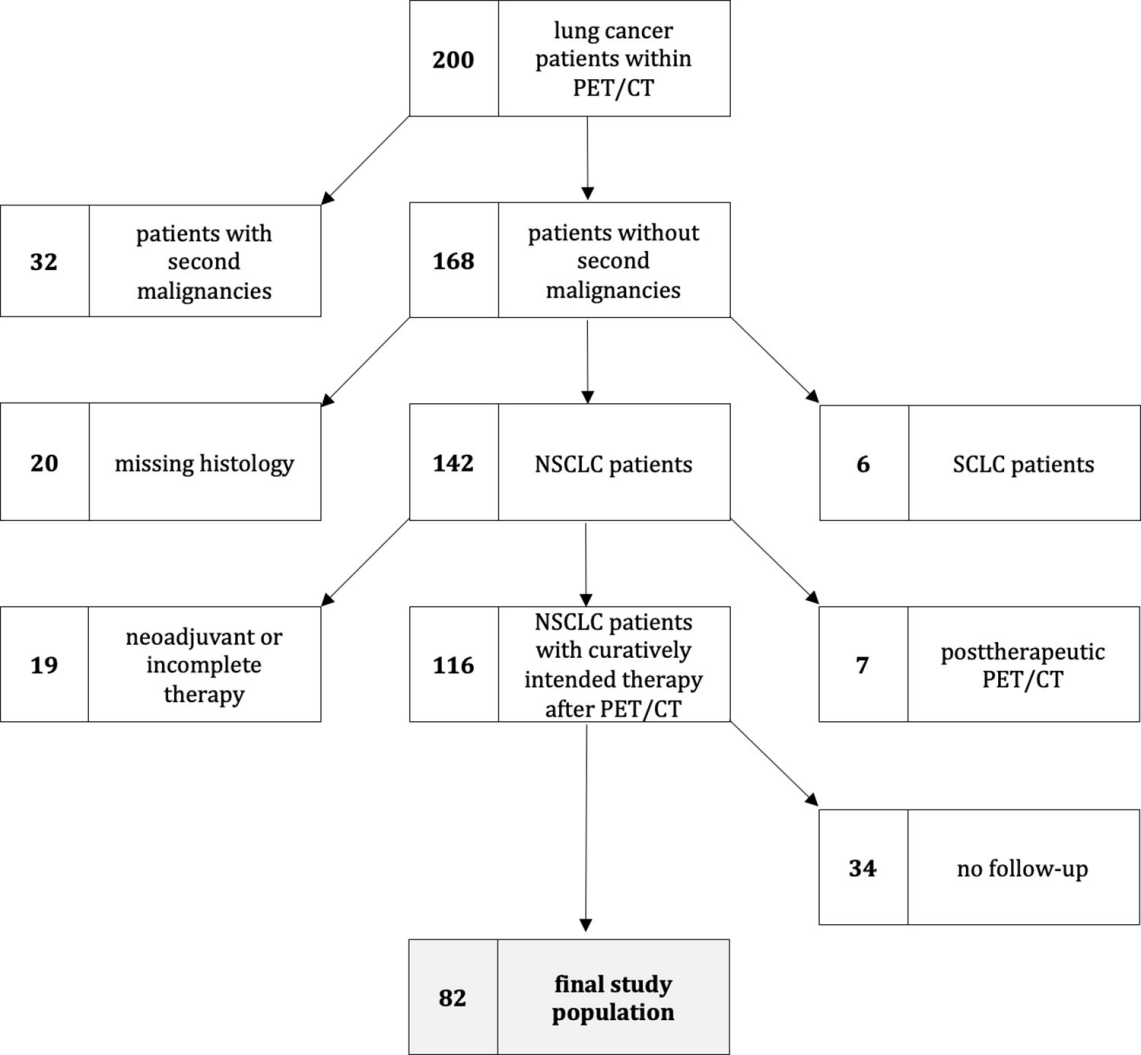
Flow-chart showing the inclusion process. The final study population consisted of n = 82 patients.

Finally, n = 82 of total 200 patients were included for further data evaluation (Fig 2) divided into 25/82 (31%) women and 57/82 (69%) men with a mean age at time of examination of 66 ± 9 years (range: 41 to 88 years).

Fifty-four of a total of 82 patients (66%) had progressive disease. A total of 17/82 patients (21%) had already died within the study period. The median follow-up time was 60 (IQR 27–106) months. Forty-nine of 82 patients (60%) suffered from adenocarcinoma, 22/82 (27%) from squamous-cell carcinoma (SCC), 6/82 (7%) from large cell neuroendocrine carcinoma (LCNEC), 3/82 (4%) from adenosquamous carcinoma (ASC), and 2/82 (2%) from non-differentiated NSCLC. The cohort consisted of 13/82 patients (16%) with stage I, 12/82 patients (15%) with stage II, 27/82 patients (33%) with stage III and 30/82 patients (37%) with a stage IV disease, respectively (Table 1).

The median tumor size was 39 (IQR 24–62) mm and the median tumor density 42 (IQR 29–52) HU. Forty-three of 82 NSCLC primary tumors (52%) had spicules. A central necrosis was detected in 43/82 (52%) lung carcinomas. The mean indexSUV_max_ of the primary tumor was 4.5 ± 2.0 (range: 0.8 to 10.7). Patients with progressive disease had a mean indexSUV_max_ of 4.5 ± 1.9 compared to 4.3 ± 2.5 for patients without tumor progression. The mean indexSUV_max_ for patients who died within the study period was 4.9 ± 0.1 compared to 4.4 ± 1.4 for patients who survived.

There was no significant difference between patients with and without progressive disease or disease-associated death with regard to indexSUV_max_ (PFS: U = 716.5, Z = - 0.386, *p* = 0.68; OS: U = 699.5; Z = -0.0870, *p* = 0.39). Using receiver operating characteristic (ROC) analysis and Youden’s index, an optimal indexSUV_max_ cut-off value of 3.8 was calculated for PFS (sensitivity 94%, specificity 75%, area under the curve [AUC] = 0.779). There was no significant difference in PFS between patients with an indexSUV_max_ > 3.8 and those ≤ 3.8 (*p* = 0.45). Patients with an indexSUV_max_ > 3.8 had a median PFS of 13 months compared to 27 months in patients with an indexSUV_max_ ≤ 3.8. Additionally, there was no significant difference in PFS between patients with central necrosis (23 months vs. 18 months, *p* = 0.64) and spicules (23 months vs. 18 months, *p* = 0.78) und those without.

Multivariate analysis revealed no significant impact of indexSUV_max_ (HR = 1.0, CI: 1.0–1.1; *p* = 0.49), tumor density (HR = 0.9; CI: 0.6–1.4; *p* = 0.73), tumor size (HR = 1.0; CI: 0.9–1.0; *p* = 0.08), central necrosis (HR = 1.0; CI: 0.6–1.8; *p* = 0.98), and spicules (HR = 1.0, CI: 0.6–1.9; *p* = 0.91) as independent predictors of PFS.

**Fig 3:**
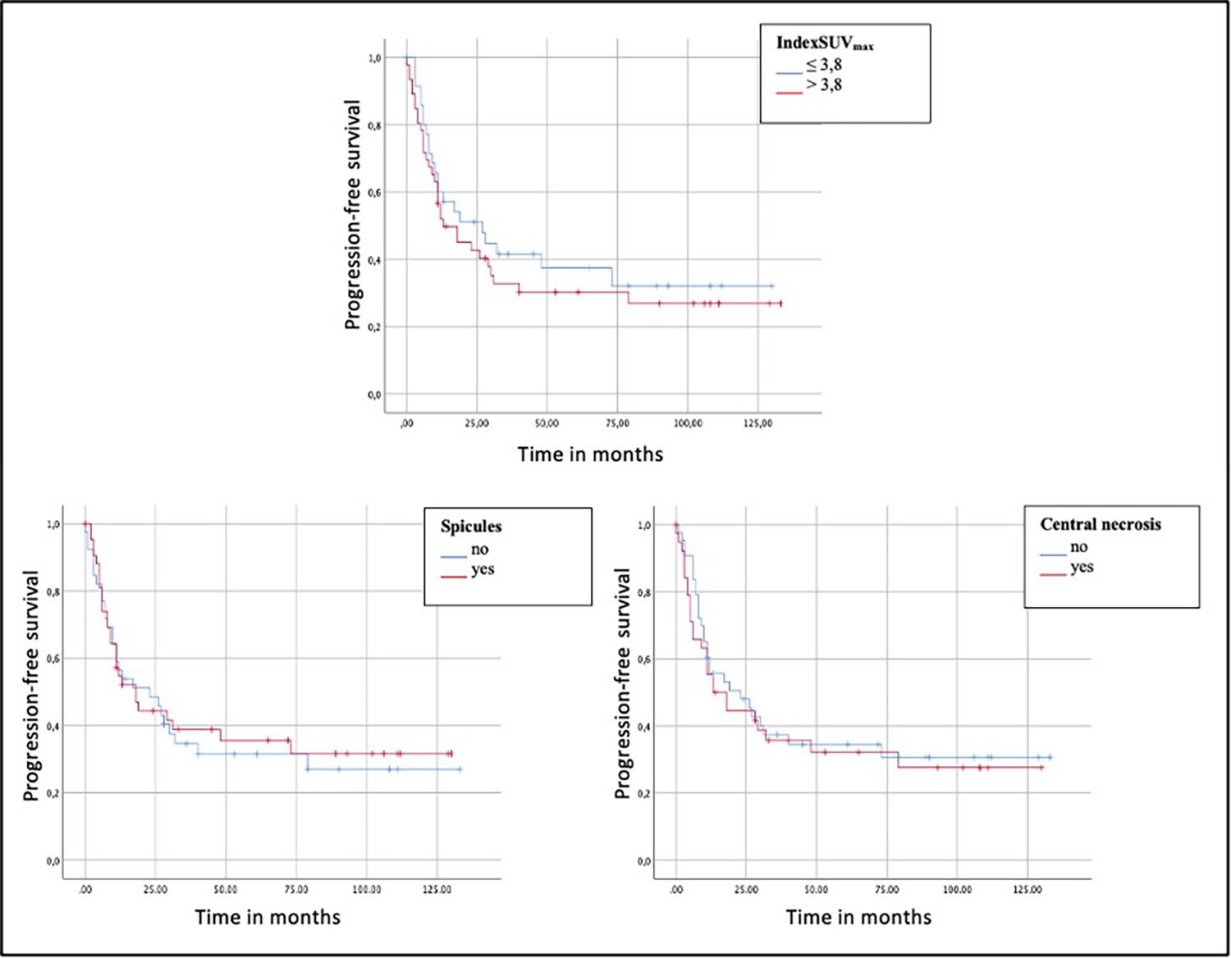
Kaplan-Meier curves of indexSUV_max_, central necrosis, and spicules. Progression-free survival (PFS) proportion against time in months are plotted in each diagram.

Kaplan-Meier curves for PFS (indexSUV_max_, central necrosis and spicules) are visualized in Fig 3.

Using ROC analysis and Youden’s index, a further optimal IndexSUV_max_ cut-off value of 4.0 was calculated (sensitivity 94%, specificity 79%, area under the curve [AUC] = 0.873) for OS. A slightly prolonged OS was documented for patients with an indexSUV_max_ of ≤ 4.0 with a mean overall survival of about 114 months compared to patients with an indexSUV_max_ > 4.0 with 106 months without statistical significance (*p* = 0.41). There was no significant difference in OS between patients with central necrosis (109 months vs. 105 months, *p* = 0.89) or spicules (109 months vs. 108 months; *p* = 0.66) and those without.

Multivariate analysis revealed no significant impact of indexSUV_max_ (HR = 1.0; CI: 0.9–1.2; *p* = 0.41), tumor density (HR = 0.3; CI: 0.1–1.1; *p* = 0.07), tumor size (HR = 1.0; CI: 0.9–1.0; *p* = 0.07), central necrosis (HR = 0.6; CI: 0.2–1.9; *p* = 0.40), and spicules (HR = 1.3; CI: 0.4–3.7; *p* = 0.65) as independent predictors for OS.

**Fig 4:**
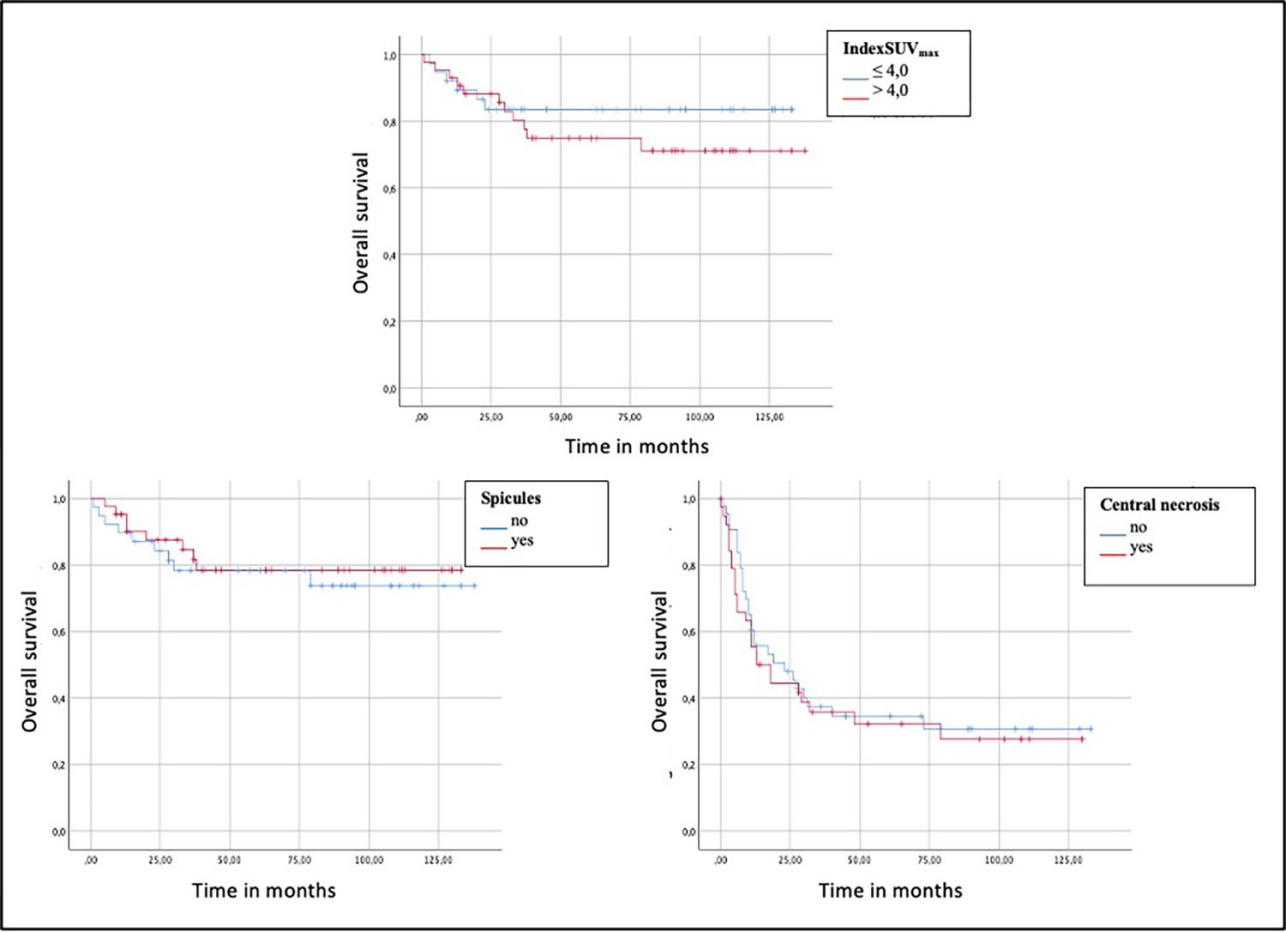
Kaplan-Meier curves of indexSUV_max_, central necrosis and spicules. Overall survival (OS) proportion against time in months are plotted in each diagram.

Kaplan-Meier curves for OS (indexSUV_max_, central necrosis and spicules) are visualized in Fig 4.

## Discussion

Metabolic and morphologic parameters from pre-therapeutic [^18^F]FDG-PET/CT were not able to predict PFS and OS in NSCLC-patients. While [^18^F]FDG-PET/CT is a central tool to define tumor stage pretherapeutically, tumor stage itself seems to remain the main factor to define patient prognosis.

The findings of this study shed light on the complexity of predicting outcomes in NSCLC-patients. Low survival rates and non-consistent treatment outcomes of NSCLC-patients make it necessary to early identify patients who are at risk for an unfavorable course of disease [2, 3]. [^18^F]FDG-PET/CT, as the imaging modality of choice for NSCLC-patients, plays a pivotal role in further treatment decisions. Its highly accurate staging accuracy enables a precise TNM and UICC staging as the most important predictor of PFS and OS by now [13]. However, a prognostic gap exists within the current staging method, indicating that patients with the same tumor stage may have different outcomes [13, 14]. Therefore, additional prognostic factors besides TNM and UICC classification are needed to identify those patients who will benefit the most from an optimized and patient-centered therapeutic regime. SUV_max_ as a metabolic [^18^F]FDG-PET/CT imaging feature has widely been investigated as a prognostic predictor for the risk of cancer recurrence and poor prognosis in various non-NSCLC cancer entities [26–28]. According to the literature, there is no clear trend for NSCLC-patients [6].

Based on the presented results, indexSUV_max_ revealed no significant prognostic impact when determining patient prognosis according to PFS and OS. The additional introduction of a diagnostic threshold of 3.8 for PFS and 4.0 for OS had no prognostic potential on survival either. Thus, a higher indexSUV_max_ of the primary tumor was not associated with a worse outcome. These results are in accordance with the work of Agarwal et al., who highlighted that preoperative SUV_max_ of LC is not an independent predictor for overall survival in patients suffering from NSCLC [18]. Similar results have been provided by Hoang et al., who did not find significant correlations between SUV_max_ of the primary tumor and patient survival [29]. In contrast, there are studies associating high SUV_max_ values with an increased risk of cancer recurrence and cancer-associated death [19–21]. For example, Sasaki et al. showed in their study that the SUV_max_ of the primary tumor was a strong predictor for PFS and OS [19]. Similar results were shown in the study of Downey et al., which pointed out that there was a significant better 2-year survival in NSCLC-patients with a SUV_max_ less than 9 [18]. In this study, unlike our data evaluation, the median SUV_max_ was defined as the diagnostic threshold, making comparability difficult. These findings emphasize the nuanced nature of SUV_max_. While it provides insights into tumor metabolism and characteristics, it does not necessarily predict prognosis in NSCLC-patients. Thus, according to the data presented, high SUV_max_ values of the primary tumor should not be strictly interpreted as poorer prognosis in the clinical diagnostic routine of NSCLC-patients. The prognostic potential of SUV_max_ of pre-therapeutic [^18^F]FDG-PET/CT for predicting PFS and OS remains controversial. Differences in study design, patient population, tumor histopathology and histopathological subtypes may account for the reported differences [6].

Moving beyond SUV_max_, CT imaging features of [^18^F]FDG-PET/CT, especially the presence of central necrosis and spicules, have been considered indicative of tumor aggressiveness and heterogenicity [30–32]. However, the prognostic potential of these features concerning individual tumor stages is not fully understood [32]. Therefore, the presented study also investigated the prognostic potential of morphological CT parameters of pre-therapeutic [^18^F]FDG-PET/CT.

Contrary to the results of our study, Wang et al. showed that the presence of spicules was an independent prognostic predictor for adenocarcinomas and patients with a spiculated primary tumor had a significantly shorter progression-free survival [31]. Also, with regard to the presence of central necrosis, there were conflicting results towards the current literature. While central necrosis of the primary tumor is considered a prognostically unfavorable factor in NSCLC [30] and consideration has already been given to incorporating this factor into TNM classification [32], this study unfortunately failed to demonstrate that central necrosis was associated with significantly worse PFS and OS. This might be based on the fact that the majority of cancer subtypes in this study are adenocarcinomas and not squamous cell carcinomas or large cell carcinomas which are associated with a higher percentage of central necrosis [30]. Furthermore, tumor size and tumor density had no significant influence on PFS and OS as well and no significant differences in tumor size and tumor density between patients with and without cancer-associated progression or -death could be revealed by group comparison. The lack of significant correlations between CT morphological parameters of [^18^F]FDG-PET/CT and PFS and OS highlights the need for caution in interpreting certain CT image morphological parameters regarding patient prognosis.

While this study contributes valuable insights, it is important to acknowledge its limitations. The main limitation of this study is the highly skewed sample with a heterogeneity of the study population. However, it should be kept in mind that the histopathologic profile of NSCLC patients consists of a wide variety of subtypes, tumor stages and therapies. The second limitation is the retrospective study design with variations of treatment protocols. Nonetheless, this reflects the clinical reality of existing data. Furthermore, the small number of patients studied is a further limitation. Unfortunately, in the context of this work patients cannot be included due to lack of follow-up, which also ultimately reflects clinical reality. Additionally, patients with neoadjuvant therapy and renouncement of therapy had to be excluded to enable a more homogenized study population. It should also be noted that only about a third of the people in the study were female, which is understandable as more men develop NSCLC. Another limitation that must be considered is that only the SUV_max_ in the primary lung lesion was measured, rather than the whole-body tumor burden. Other metabolic parameters, such as metabolic tumor volume (MTV) and total lesion glycolysis (TLG), are certainly relevant in this context. However, the focus of this study is on SUV_max_, as it is a clinically applicable and easily determinable parameter with wide acceptance in clinical practice.

Although the limitations may reflect clinical reality, it also supports large-scale prospective studies at multiple centers to address such problems and acquire homogeneous, large data sets. The purpose is to set a clear trend of the prognostic value of SUV_max_ and other imaging markers of a pre-therapeutic [^18^F]FDG-PET/CT. These large data would also be able to be implemented artificial intelligence (AI) tools to represent correlations beyond conventional statistics. It would be interesting whether AI analyses are able to identify prognostic predictors of [^18^F]FDG-PET/CT.

## Conclusion

Metabolic (SUV_max_) and morphologic parameters from pre-therapeutic [^18^F]FDG-PET/CT were not able to predict PFS and OS in NSCLC-patients.

## Supporting information

S1 Graphical abstract (TIFF)
