# Supplementary figures and images for: Prognostic potential of integrated morphologic and metabolic parameters of pre-therapeutic [18F]FDG-PET/CT regarding progression-free survival (PFS) and overall survival (OS) in NSCLC-patients
Source: PLoS One. 2024 Jul 29;19(7):e0307998. doi: 10.1371/journal.pone.0307998 (PMC11285944; doi:10.1371/journal.pone.0307998)

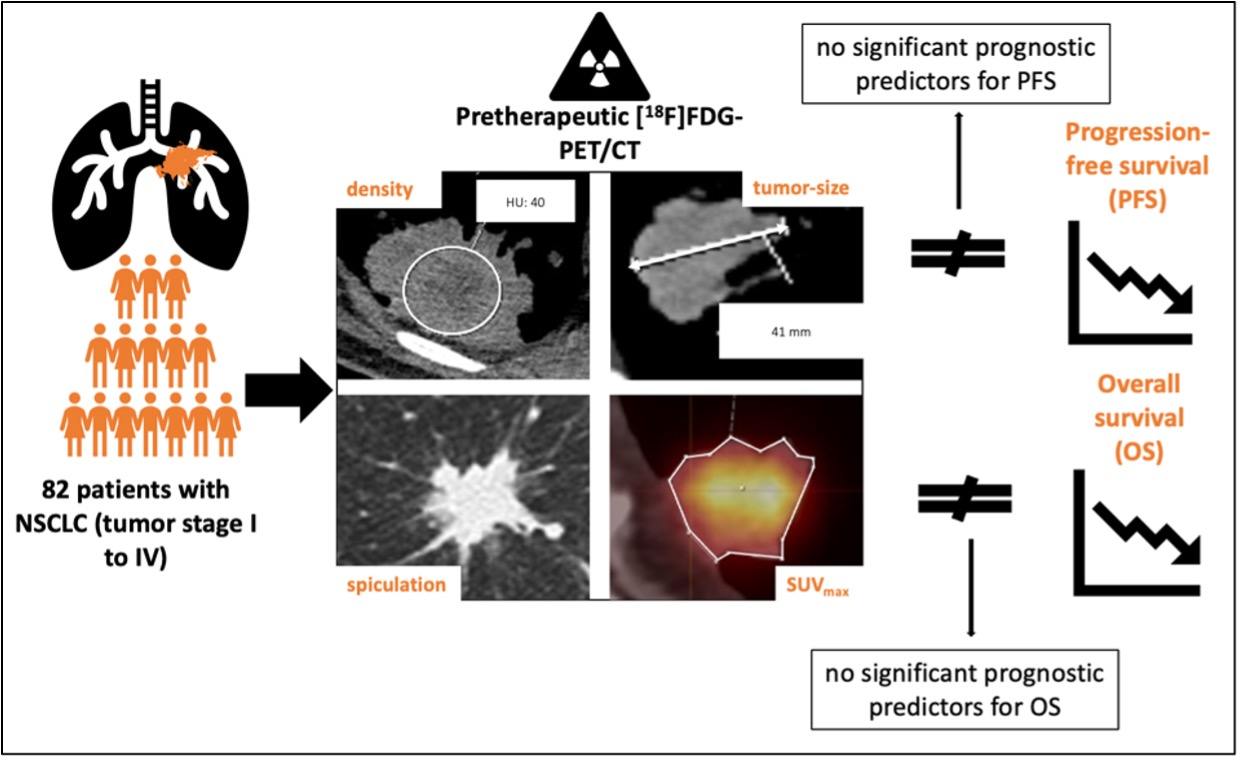

Supplement: S1 Graphical abstract — (TIFF) [file pone.0307998.s001.tiff]
